# Adults with Intellectual Disability and Autism Spectrum Disorder: What Is the Evidence around the Use of Polypharmacy

**DOI:** 10.3390/ijerph192315974

**Published:** 2022-11-30

**Authors:** Jane M. McCarthy, Eddie Chaplin

**Affiliations:** 1 Faculty of Medical and Health Sciences, University of Auckland, Auckland 1023, New Zealand; 2 Department of Forensic and Neurodevelopmental Sciences, Institute of Psychiatry, Psychology & Neuroscience, King’s College London, London SE5 8AF, UK; 3 Foundation for People with Learning Disabilities, Institute of Health and Social Care, London South Bank University, London SE1 0AA, UK

**Keywords:** autism spectrum disorder, comorbidity, intellectual disability, psychopharmacology, polypharmacy, prescribing

## Abstract

A review on the mental health needs of adults with intellectual disability (ID) and autism spectrum disorder (ASD) published just over 10 years ago found a limited evidence base for pharmacological intervention in this group. The aim of this paper was therefore to review the evidence in the subsequent 10 years, with a focus on polypharmacy use in adults who have both ID and ASD. A critical literature review of key papers published from 2009 to 2021 was undertaken on adults with both ID and ASD and related to psychopharmacology, polypharmacy, antipsychotics, antidepressants, mood stabilisers and anxiolytics interventions in improving symptoms. After excluding articles for lack of relevance, a review with a focus on the use of polypharmacy was carried out on the retrieved results. Four papers were identified as relevant to adults with both ID and ASD. Three main themes were identified in the review, including the application of pharmacogenetics, the influence of national policy on prescribing practices and safety concerns in a population with multiple health comorbidities. The past decade has produced a small increase in the evidence base on psychopharmacology use in adults with ID and ASD. However, more evidence on the effectiveness and impact of long-term polypharmacy use is required.

## 1. Introduction

This is a review focusing on the group of adults with intellectual disability (ID) and autism spectrum disorder. It is an update to a review just over 10 years ago that found a limited evidence base for pharmacological interventions in this group [1]. Polypharmacy is the use of multiple medications and is normal practice when treating multimorbid cases and when managing single diseases. Guidelines on specific diseases are associated with a rise in the use of polypharmacy [2]. For adults with intellectual disability, there have been concerns about adverse effects and the inappropriate use of polypharmacy within psychiatric settings [3]. Rates of over 40% for polypharmacy use in adults with ID who exhibit aggressive behaviour are reported [4]. The risks and benefits of combining different psychotropic medication in those with ID and challenging behaviour are unknown [5]. In addressing this, we also need to be mindful that the use of more than one psychotropic medication may also bring benefits when used in the appropriate clinical context. This occurs across psychiatric practice, for example, with the use of aripiprazole for the augmentation of antidepressant-refractory depression [6].

The co-occurrence of ID in autism spectrum disorder (ASD) was previously estimated to be between 50% and 70%, although more recent reports suggest the figure to be around 20%, with higher prevalence of ASD in people with more severe ID [7]. It is well recognised that adults with autism have increased rates of psychotic disorder and bipolar disorder. However, differentiating the core symptoms of autism from the core symptoms of psychiatric disorder can be challenging and difficult at times, especially for those with ID [8,9]. A diagnosis of psychopathology can be made more difficult given the symptomatic overlap between ID and ASD and the lack of standardised measures for diagnosing these conditions. The presence of challenging behaviour and the association with psychiatric disorders in those with ID is complex. A comparison study of problem behaviour (PB) in Major Depressive Disorder (MDD), in which two groups with and without PB, with mild-to-moderate ID and ASD, respectively, found that the prevalence of MDD was significantly higher in the problem behaviour group. This suggests that MDD can coexist or appear where there is an exaggeration of problem behaviour in people with ID [10]. A more recent community study from Spain found that underdiagnosed mental disorders in adults with ID and ASD were high and associated with challenging behaviour [11]. However, an earlier outpatient/clinic-based study in London of adults with ID did not find a relationship between challenging behaviour and psychiatric disorder in those with ASD [9]. 

A review on the mental health needs of adults with both ID and ASD published over ten years ago found that those who were more vulnerable to psychiatric disorders also had more impaired social skills. Interventions to improve adaptive skills needed further study. A clinic study [12] found that the main problem behaviours in those with both ID and ASD were not anxiety or depressive symptoms, but rather communication disturbance, poor social relations or being socially absorbed. This indicates that the development of skills around social and communication should be the focus in this group, rather than the use of psychotropic medication. 

Good quality evidence for the use of psychotropic medication in adults with ASD is limited. Recommendations are largely based on studies from children [13], with the most evidence for the use of risperidone and aripiprazole for irritabilities and problem behaviours [14]. However, a study published over a decade ago [15] found that in adults with ID, a placebo was more effective than either risperidone or haloperidol. There are no published random controlled trials for the use of Selective Serotonin Reuptake Inhibitors (SSRIs) in the treatment of anxiety, depression and obsessional compulsive disorders in adults with autism [16].

Despite this lack of evidence, psychotropic medication prescription rates of 30 to 90% for those with ID and challenging behaviour have been reported [17]. It is in this context that the national STOMP policy (STop Over Medication in People with intellectual disability and autism or both) in England (www.england.nhs.uk/stomp) [Accessed on 25 October 2022] was established. STOMP was planned as a three-year programme by the National Health Service in England. A review [18] describing the background of this initiative found that despite many studies and guidelines, the use of psychotropic medication in people with ID and autism remains common. The aim of STOMP is to achieve a significant reduction and reliance on psychotropic medication in people with ID and ASD by ensuring that alternative and more appropriate psychologically focused interventions are the first choice of treatment. It also aims to coordinate such treatment with interventions such as sensory adaptations to reduce the long-term use of pharmacological medication. However, it is still the case that there are no randomised controlled studies that test the efficacy of combinations of interventions, including the use of psychopharmacology in combination with behavioural and sensory interventions. 

In addition, there is a lack of research to inform clinical practice comparing different medication combinations, specifically the ongoing use of polypharmacy in adults with both ID and ASD who present with problem behaviours. Polypharmacy is recognised in day-to-day practice to occur for adults with ASD as well as those with ID presenting to psychiatric services. 

A study from Spain of 66 adults with ASD by [19] found the presence of ID to be a significant predictor of polypharmacy (defined in the study as the use of two or more psychotropic drugs in the same individual). Another study [3], which sought to identify predictors of psychotropic polypharmacy in those with ID, audited the prescribing practice for 517 adults with ID who were referred to a specialised psychiatric outpatient clinic between 2005 and 2013. They found that 22% of the patients were receiving psychotropic polypharmacy. Women, those living in supervised residential settings and those with a psychiatric diagnosis in two or more diagnostic categories were more likely to receive psychotropic polypharmacy. The study concluded that polypharmacy is a significant concern for adults with an ID seeking specialised psychiatric services, and that there are multiple contributing factors to this prescribing practice. 

The focus of this paper is therefore on adults with ID who also have ASD. This paper neither focuses on autistic adults who do not have significant impairment of intellectual functioning nor on adults with ID who are not autistic. The key aim of this paper is to present the evidence from the past decade on the use of polypharmacy in those who have both ID and ASD. From the limited available evidence, this seems to be the group of patients who are most at risk of being prescribed more than one psychotropic medication. The aim was also to understand the clinical rationale for this practice and whether this practice is monitored, as there is no evidence to support this practice in adults with both ID and ASD. Although only ten years have passed since the last review, this group remains poorly understood. Many patients exhibit behaviours that pose a risk to themselves or others that present significant cost and resource challenges for services and commissioners. Another review from the United States undertaken over a decade ago concluded that the effectiveness of psychotropic medications in managing challenging behaviours in people with ID was minimal and called for controlled studies of higher scientific quality in this area [20]. Current pharmacological interventions used in clinical practice may not achieve their intended outcome and have unintended effects that may affect the overall wellbeing of the individual. Polypharmacy is one such example which can have several negative outcomes, including an increased risk for side effects such as metabolic side effects and harmful drug–drug interactions. However, polypharmacy may be clinically recommended in high-risk scenarios of problem behaviours, comorbid conditions or in scenarios when augmentation of medication may be prescribed.

## 2. Materials and Methods

The aim of the review was to examine outcomes of pharmacological interventions in adults with both ASD and ID as relevant to clinical practice with a focus on polypharmacy. The following databases were searched to identify key papers published from 2009 to 2021: Medline, Embase, PsycINFO and Cochrane Library. The search was conducted by the British Medical Association Library in February 2022. The PICO (Population, Intervention, Comparison and Outcomes) framework was used to inform the search. PICO is a recognised framework updated in 2022 and used in evidence-based practice to formulate a review question on the effectiveness of an intervention [21]. Please see Table 1.

### Inclusion and Exclusion Criteria

The inclusion criteria for the review were:

- Adults over the age of 18 years old;
- Research conducted between 2009 and 2021.

The exclusion criteria were:

- Single case studies;
- Articles not written in English.

**Figure 1:**
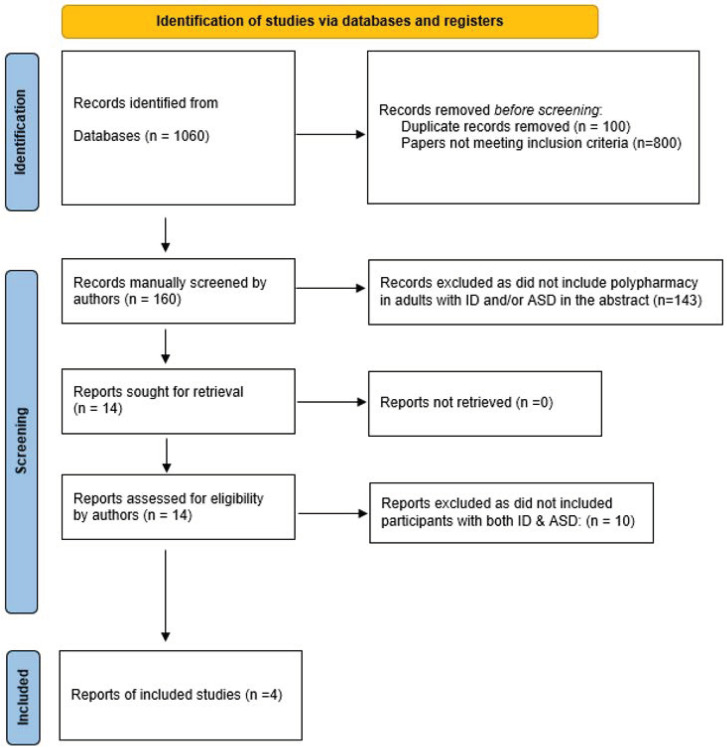
Prisma systematic review flow diagram.

The search was conducted in the British Medical Association (BMA) library. It produced 1060 results. After removing duplicates and following a manual sifting for relevance to the review, 160 papers were identified. The abstracts of all 160 papers were screened by the authors. Of these, 14 papers mentioned polypharmacy in adults with ID and/or ASD in the abstract. Ten of these fourteen papers did not include adults with both ID and ASD; leaving four papers that were eligible to be included in the review. Please see Figure 1.

## 3. Results

### 3.1. Current Review

The four papers reviewed (see Table 2) focused on three themes.

1. Pharmacogenetics [22];
2. Trends in Prescribing [23];
3. Multimorbidity and Patient Safety [24,25];

**Table 2:** Summary of findings from identified papers reviewed.

No.	Author, Year	Participant Information	Location & Methods	Main Findings
1	Yoshida, K. et al. (2021) [21].	28 (Pharmacogenetics) PGx studies were identified through a systematic review. Three studies included adults with ASD/ID. The other studies focused on children and adolescents with ASD/ID.	A systematic review paper from Canada	There is limited data available from PGx studies in individuals with ASD/ID and in particular in adults. Given the potential for PGx testing in improving treatment outcomes, additional pharmacogenomic studies for psychotropic treatment in ASD/ID across age groups are warranted.
2	Espadas, C. et al. (2020) [24].	Adults with ASD-ID (n = 83).	An observational, multicentre pharmacovigilance study in Spain of patients diagnosed with ASD and ID.	Risperidone and quetiapine were co-prescribed in 60% of the cases without any monitoring of adverse events being routine. The rates of multimorbidity and polypharmacy, among young adults with ASD and ID, were concerning.
3	Miot, S. et al. (2019) [25].	Adults with ASD-ID (n = 63).	Detailed clinical examinations from nine specialist institutions in France.	A large range of comorbidities, gastrointestinal disorders and mental and neurological diseases were identified. Overall, 25% of ASD-ID sample had chronic kidney disease with associated increased cardiovascular risk factors. The comorbidity burden was high and comparable with that observed among older hospitalised patients in geriatric departments. Furthermore, the comorbidity burden positively correlated with age, decreased autonomy, and polypharmacy.
4	Mehta H & Glover G (2019) [23].	Not stated.	A quantitative, descriptive study using data from The Health Improvement Network (THIN) database of Primary Care Practices in England.	Analyses looked separately at adults with ID and autistic children and young people. For adults with ID, favourable changes in trend were seen for the overall prescribing of antipsychotics, antidepressants and anxiolytics, but further analysis concerning the presence of recorded appropriate clinical indications produced a complex picture. Some measures of polypharmacy showed improvement.

#### 3.1.1. Research Methods 

The research methods employed within the included studies used a mixture of review, observational, clinical examination and existing databases. Though these may not be the most robust methods, they reflect the emergence of this area of research and identify potential issues that relate to interventions and research methods for future studies, which are discussed below.

#### 3.1.2. Potential of Pharmacogenetics

Pharmacogenetics is the study of the influence of genetic factors on the efficacy and tolerability of medication. Though the evidence around the use of antipsychotics has grown in recent years, the application to practice has been limited. A systematic review [22] examined pharmacogenomic (PGx) studies in those with ASD and/or ID (ASD/ID). In particular, PGx studies of antipsychotics and antidepressants analysing treatment response and adverse effects in those with ASD/ID were studied. A total of 28 PGx studies using mostly candidate gene approaches were identified across age groups. Notably, only three studies included adults with ASD/ID. The other 25 studies focused specifically on children/adolescents with ASD/ID. Although the study reported interesting findings, it illustrated that there are limited data available on PGx in individuals—in particular, in adults—with ASD/ID. However, two sets of gene polymorphism showed potential for further study. Given the potential for PGx testing in improving treatment outcomes, The authors recommended that additional PGx studies on the use of psychotropic treatment in ASD/ID across age groups are warranted.

#### 3.1.3. Trends in Prescribing in Response to National Policy

A study [23] was built on the findings on the prescription of psychotropic medication by general practitioners (GPs) to people with ID. This led the NHS in England to initiate a programme to reduce inappropriate prescription of medication, known as the STOMP Policy, described earlier. This current paper was an observational study to monitor progress and used anonymised research data collected from general practices in England. The study analysed trends in prescription prevalence and quality. For adults with ID, favourable changes in trend were seen for the overall prescription of antipsychotics, antidepressants and anxiolytics, but further analysis in relation to the presence of recorded appropriate clinical indications produced a complex picture. Before the STOMP launch, rates of within-group polypharmacy showed falling trends for antipsychotics and antiepileptics for adults with ID. This trend did not change significantly post-STOMP. The rate for antidepressants initially showed a rising trend but this changed to a flatter trend post-STOMP. Between-group polypharmacy showed a rising trend pre-STOMP which changed to a falling trend post-STOMP.

#### 3.1.4. Multimorbidity and Patient Safety

A multicentre study [24] from Spain aimed to evaluate medication-related safety, drug–drug interactions and psychotropics prescription trends. This was an observational pharmacovigilance study of 83 subjects with ASD and ID. Risperidone and quetiapine were co-prescribed in 60% of the cases. A lack of monitoring of adverse events was routine. The study showed concerning rates of 37% for multimorbidity and 57% for polypharmacy in young adults with ASD and ID. The authors suggested the need to develop a pharmacovigilance monitoring system to evaluate the long-term safety of ongoing use of psychotropic medication in the autistic population with associated ID. A study from France [25] aimed to determine the comorbidity burden and its association with distinct clinical presentation in terms of ASD severity, adaptive skills, level of autonomy and drug exposure in a well-phenotyped sample of individuals with ASD-ID. Sixty-three adults with ASD-ID were recruited from 2015 to 2017 from nine specialized institutions in France. Detailed clinical examinations were undertaken, including screening for comorbidities, ASD severity, adaptive functioning, autonomy and drug use including polypharmacy. The study found a large range of comorbidities, including gastrointestinal disorders, mental disorders and neurological diseases. The comorbidity burden positively correlated with polypharmacy use as well as with age and decreased autonomy. The authors highlighted the need for personalised medical care in this group of patients.

## 4. Discussion

This review highlights the very limited evidence available for the outcome of polypharmacy practice in adults with both ID and ASD.

There were only four papers identified by the current review as relevant to adults with both ID and ASD. Three main themes were identified, including developments in pharmacogenetics, the influence of national policy on prescribing practices and safety concerns in a population with multiple health comorbidities. The studies ranged from examining the potential improvement of treatment outcomes using pharmacogenomics to the analysis of clinical practice data across a number of European countries, including France, Spain and the UK. It appears that although there has been a positive trend toward the prescription of psychotropic medication—for example, as influenced by the national policy in England—further analysis is still required to study the presence of clinical outcome indicators to warrant the use of such medication. A recent study indicates that in many locations across England, antidepressants have become the most widely prescribed psychotropic for adults with ID. However, the clinical indication for this change in practice is unclear [18]. Many individuals with ID and ASD will have other co-existing physical and/or mental conditions, adding to the complexity of decision making around prescribing medication. Problems may include ensuring an accurate diagnosis with the risk of diagnostic overshadowing and psycho-social masking to potential contra-indications with any current treatments. The evidence from the papers reviewed suggests the use of two or more psychotropics remains high. 

Other papers relevant to the wider debate on the use of psychotropic medication in adults with ASD and ID, that may help to inform future research studies, were also reviewed. These included Sheehan and colleagues [26], who investigated the incidence of new prescriptions of psychotropic medication to people with ID in the UK aged 18 years and older, and used population-level data from 571 general practices. The data showed that challenging behaviour or autism was independently associated with a new prescription of all antipsychotic medication. This indicates that autism is a risk factor for receiving psychotropic medication in adults with ID. 

A review of the use of psychotropic medication in those with low functioning autism [8] reported that psychotropic medication was mainly used for those presenting with high-risk behaviours. The most commonly prescribed medications were second-generation antipsychotics, with risperidone and aripiprazole being used for irritability, aggression, self-injury and severe tantrums. Less promising results for SSRIs and mood stabilisers were shown. The review concluded that psychotropic medication should only be used when non-pharmacological strategies fail as part of a multidisciplinary team assessment and the evidence on efficacy, dosage and safety is scarce.

A systematic review [27] of the evidence for treating challenging behaviours using psychotropic medication in adults with autism and ID identified seven articles. Five agents were included in these seven articles: fluvoxamine, sertraline, clomipramine, risperidone and ziprasidone. Randomised control studies of fluvoxamine and risperidone showed efficacy for the treatment of challenging behaviour in adults with autism and ID. There is therefore a very small evidence base which supports the use of psychotropic medication in adults with autism and ID.

In one study, 225 UK psychiatrists working in the field of ID were surveyed using an online questionnaire [28]. Eighty-eight responded with complete antipsychotic withdrawal in over 50% of patients treated, with antipsychotics being achieved by only 4.5% of respondents. Factors hampering withdrawal include concerns of family/paid carers, lack of multiagency and multidisciplinary input and non-availability of non-medical psychosocial interventions. This compares with a 40% success rate for 129 individuals specifically identified for discontinuation in a Dutch study [29] and a success rate for withdrawing antipsychotics of 46.5% in Cornwall, England using a structured pathway among 77 adults with ID living in the community [30]. 

Indications for the use of polypharmacy is limited by this lack of evidence on the efficacy of the wider group of psychotropic medication in adults with both ID and ASD. 

The clinician is therefore left with the challenge of identifying which psychotropic medication may work for an individual patient and seeking combinations of medications when there is a limited response to one psychotropic. In addition, as the evidence above shows, patients face challenges withdrawing from any psychotropic medication after initially beginning such medications, and thus require a multi-disciplinary and multi-agency approach. The review identified a need for prospective studies that focus on efficacy and safety given the current concerns. There are two issues, firstly, to ensure prescription practices are safe and appropriately monitored and secondly, to determine if there is evidence for the use of polypharmacy in specific cases. Predictors of psychotropic use include having autism, age and lower functioning. However, predictors can also be behavioural and can include agitation, aggression and self-injury. 

The development of guidelines, national policy and research are key to improving practice in this area as highlighted in a recent study by [23]. While there are national guidelines on the use of psychotropic medication in adults with ID [31], none specifically focus on polypharmacy use in adults with both ID and ASD. Research must develop around both the methodology and use of polypharmacy in practice. A review of polypharmacy focusing on the elderly and people with ID identified significant variability in methods used to study polypharmacy, including definitions of polypharmacy, samples studied, analytic strategies and variables included in the analyses [32]. Four factors to improve research in this area were identified as follows: the use of consistent definitions of polypharmacy, the implementation of population-based sampling strategies, the development of clinical guidelines and the importance of studying associated variables. In addition, calls for future research in this area must include studies on the benefits and risks of specific combinations of psychotropic medication. For example, the study by Espadas and colleagues [24] found that 60% of patients were being prescribed risperidone and quetiapine as a combination. When more evidence is available through robust studies on specific combinations of psychotropic medication, this will lead to a meta-analysis study which compares the benefits and risks of specific combinations of pharmacological interventions.

The recently published Lancet Commission on the future care and clinical research in autism proposes a modified step-care approach with a personalised mode of intervention and assessment for individuals with autism and their families [16]. The commission emphasises that any interventions or assessments must take the context of the individual, their family, cultural and regional diversities into account. It may be that advances in pharmacogenetics will support a personalised approach to the use of psychotropics, including combinations of psychotropic medication. However, as clinicians, we must continue to assess situations using a person-centred approach. Such approaches should take how each individual responds or not to a specific psychotropic medication into account, in the absence of clear evidence on optimal doses.

### Limitations

Given the relatively small size of the papers identified for this review, the conclusions will be limited to the effectiveness of polypharmacy in adults with both ASD and ID. The review accessed databases to identify papers. A search engine such as Google Scholar may have identified other papers. The criteria for inclusion were set to be very focused on only adults with both ID and ASD as an attempt to delineate the population under study and to be relevant to the psychiatrists who see this population in their day-to-day clinical practice. However, this brief review has highlighted emerging themes around patient safety and the need for a more personalised approach to treatment. The overall lack of evidence is probably a reflection of developing good quality research within this population. Tyrer et al. [15] found several challenges to examining the ongoing use of combinations of medications, including polypharmacy, in a group of patients with autism and intellectual disability in their randomised controlled trial. The current lack of evidence has highlighted the need to look at other ways to study interventions that can be evaluated beyond the traditional standards [16]. There are studies in progress that use a multicentre double-blind placebo-controlled randomised trial to study the withdrawal of antipsychotic medication in adults with ID [33].

## 5. Conclusions 

To enhance both future research and practice, there is a need for specific clinical guidelines on the use of polypharmacy to ensure a consistent approach and to develop an awareness of the needs of this group. The benefits of polypharmacy, including the impact of long-term polypharmacy and specific combinations of psychotropic medications, require further evaluation. Over time, it is possible that specialist clinics can develop good practice guidance around complex regimes of polypharmacy to increase the evidence base. It is also possible to implement a shared database that collates current practice in the use of polypharmacy for adults with ID and ASD across Europe. 

## Figures and Tables

**Table 1:** PICO Search strategy.

Population	Autism Spectrum Disorder (ASD) and Intellectual Disability
Intervention	Psychopharmacology antipsychotics, antidepressants, mood stabilisers, anxiolytics, Polypharmacy
Comparison	High-functioning ASD
Outcome	Symptom improvement, Remission of psychiatric disorder, Quality of life

## Data Availability

Not applicable.
